# Prevalence and correlates of intestinal schistosomiasis infection among school-aged children in North-Western Tanzania

**DOI:** 10.1371/journal.pone.0228770

**Published:** 2020-02-05

**Authors:** Rajabu Hussein Mnkugwe, Omary S. Minzi, Safari M. Kinung'hi, Appolinary A. Kamuhabwa, Eleni Aklillu

**Affiliations:** 1 Department of Clinical Pharmacology, School of Medicine, Muhimbili University of Health and Allied Sciences, Dar es Salaam, Tanzania; 2 Division of Clinical Pharmacology, Department of Laboratory Medicine, Karolinska University Hospital-Huddinge, Karolinska Institutet, Stockholm, Sweden; 3 Department of Clinical Pharmacy and Pharmacology, School of Pharmacy, Muhimbili University of Health and Allied Sciences, Dar es Salaam, Tanzania; 4 National Institute for Medical Research (NIMR), Mwanza Research Centre, Mwanza, Tanzania; George Washington University School of Medicine and Health Sciences, UNITED STATES

## Abstract

**Background:**

Schistosomiasis is a neglected tropical disease that continues to cause morbidity and mortality in Sub Saharan Africa. Due to its endemicity, co-infection with malaria is common. The diseases cause anaemia and impaired nutritional status among children. We investigated the prevalence of intestinal schistosomiasis and its association with malaria, anaemia and nutritional status among school children.

**Methods:**

This was a cross sectional survey among 830 children in Nyamikoma village along Lake Victoria in Tanzania. A pre-tested questionnaire was used to collect socio-demographic data, history of drug use, and clinical data. Two faecal samples were collected on two consecutive days and analyzed using thick smears Kato Katz method. Diagnosis of malaria was done by malaria rapid diagnostic test, and haemoglobin concentration was determined using HemoCue. Nutritional status was assessed by anthropometric measurements.

**Results:**

The overall prevalence of intestinal schistosomiasis was 90.6% (95% CI = 88.6% - 92.6%). Intensity of infection was light 24.1% (200/830), moderate 38.4% (319/830) and heavy 28.1% (233/830). Pre-adolescents (≤12 years) were more infected with intestinal schistosomiasis (93.2%) than adolescents (>12 years) (84.7%) (*p* < 0.001). Prevalence of malaria was 1.7% (14/824), and that of intestinal schistosomiasis–malaria co-infection was 1.6% (13/824). The overall prevalence of anaemia was 24.6% (95%CI = 18.7% - 30.5%). Severe anaemia was found in 2.3% (19/824) of study participants. The prevalence of stunting and wasting were 29.0% and 11.3%, respectively. On both univariate and multivariate regression analysis, only lower age was significantly associated intestinal schistosomiasis infection, but not anemia, malaria, stunting or wasting. However among those infected, a negative binomial regression analysis indicated independent significant association of male sex, loose stool consistency, and stunting with high eggs count/gram of stool.

**Conclusions:**

Despite several rounds of annual mass praziquantel administration, intestinal schistosomiasis is highly prevalent among school children particularly in younger children living in the study area. Biannual targeted mass praziquantel treatments or alternative regimens may be considered in future in the study area to redress the situation.

## Introduction

Schistosomiasis is a Neglected Tropical Disease (NTD) that continues to cause morbidity and mortality to many people [1]. Schistosomiasis is caused by a parasite of the genus Schistosoma. There are two species of major public health importance in Sub Saharan Africa (SSA), namely *Schistosoma mansoni* and *Schistosoma haematobium*, which cause intestinal and urogenital schistosomiasis, respectively [2]. Tanzania is ranked second to Nigeria in terms of schistosomiasis burden among the African countries [3]. In Tanzania, both urogenital schistosomiasis (*S*. *haematobium*) and intestinal schistosomiasis (*S*. *mansoni*) are endemic throughout the country, with the latter being highly prevalent around the Lake Zone where a prevalence of up to 100% have been reported [4].

Among the parasitic diseases, schistosomiasis ranks second to malaria in terms of number of cases and those at risk [3]. Globally, it is estimated that 779 million people at risk, and more than 207 million people infected with schistosomiasis worldwide [3, 5]. Apart from its health consequences, schistosomiasis is associated with negative social and economic impacts [5]. In SSA, despite ongoing control interventions, schistosomiasis contributes to more than 90% of the global schistosomiasis burden [6]. Schistosomiasis causes a loss of about 4.5 million Disability-Adjusted Life Years (DALYs) and 150,000–280,000 deaths annually in the region [5].

The disease mostly affects school-aged children in low-income communities, but all age groups can be victims due to different water contact activities such as fishing and domestic water use [1]. Infected children have poor school attendance, poor concentration in class and poor academic performance in school either due to illness itself or associated morbidities such as anaemia, fatigue, poor growth and poor cognitive development [1, 7]. For adults, the infected become less productive to their families and communities.

Like other NTDs, schistosomiasis is linked to the Sustainable Development Goals (SDGs); and its control is associated with direct impact on achieving the goals; such goals include reduction of poverty (SDG1), ensure healthy lives and promote well-being for all at all ages (SDG3) and attaining quality education to children (SDG4) [5, 8–10]. A study done in Kenya, reported an increase in school participation among school children and an increase in productivity among workers who had a history of receiving mass praziquantel treatments while in school [11]. Due to their global burden and impact on development, in 2015 the United Nations General Summit recognized and gave special attention to NTDs under the list of SDGs, Goal number 3.3: “ending the epidemic of NTDs by 2030” [12]. Schistosomiasis is among the 17 NTDs earmarked for elimination globally [13]. Apart from delaying the achievement of the SDGs, NTDs such as schistosomiasis are associated with a negative impact on core components of the human development index (HDI), which include the standard of living, educational attainment and years of schooling, and years of life lived with good health [14].

The World Health Organization (WHO) promotes five main public health interventions against schistosomiasis; which include preventive chemotherapy, vector control, access to safe and clean drinking water, basic sanitation and hygiene services, and health education [15]. In Tanzania, the use of mass praziquantel treatments as preventive chemotherapy was initiated in 2004–2005 and is the main strategy for schistosomiasis prevention and control targeting school-aged children. By 2016, Tanzania had reached 100% geographical coverage in terms of praziquantel mass drug administration targeting school-aged children.

Malaria continues to be a disease of the global concern contributing to 445,000 deaths annually [16]. In African countries, malaria is reported to account for between 13% and 50% of the medical reasons for school absenteeism among school children annually [17]. In Tanzania, the trend of malaria prevalence has increased from 9% in 2011–2012 to 14% in 2015–2016 with the high prevalence reported around the Lake Zone [18]. The Lake Zone is one of the high transmission areas for malaria in the country where a prevalence of more than 40% has been reported [18]. Like in schistosomiasis, when the prevalence and burden of malaria increase, children are more affected.

Due to their endemicity, geographical overlap, and presence of water bodies (breeding site for vectors of both diseases), co-infection of malaria and schistosomiasis is common [19]. Co-infection of the parasitic infections of schistosomiasis and malaria results in more severe and prolonged morbidity especially in children [20, 21]. Therefore, a regular assessment of the prevalence of the diseases, and diseases-associated negative health outcomes such as anaemia and impaired nutritional status especially among school children is necessary to inform the national diseases control programs. With regard to the reported high prevalence of *S*. *mansoni* in previous studies around Lake Zone in Tanzania but also the fact that praziquantel has been reported to be more efficacious against *S*. *haematobium* [22] than *S*. *mansoni* [23] therefore, this study was focused on assessing *S*. *mansoni* epidemiology. We investigated the prevalence of intestinal schistosomiasis, and its association with malaria, anaemia, and undernutrition, if any, among school-aged children residing in a rural area along the shores of Lake Victoria in North-Western Tanzania.

## Materials and methods

### Study setting and population

The study was conducted in Busega district, Simiyu region, North-Western Tanzania between February and May 2017 in collaboration with the National Institute for Medical Research (NIMR) Mwanza Research Centre, Tanzania. Busega district is located along the Lake Victoria basin and borders the Lake to the North. The district receives two round of rainfall per year, light rains around October to December and heavy rains around March to May of every year. The average annual rainfall ranges from 700 mm to 1000mm. The mean temperature in the district ranges between 18⁰C to 20⁰C during rainy season and up to 32⁰C during dry season. Transmission of both schistosomiasis and malaria infections is facilitated by the presence of rainfall. The two diseases are among the top 10 causes of morbidity and mortality in the district. The district had received five rounds of mass praziquantel treatments in 2005, 2007, 2013, 2014 and 2015. No mass praziquantel treatment was administered in 2016, a year before data collection for this study was done. The national treatment coverage for mass drug administration has increased from 77% in 2015 to 90% in 2016 (4.71 million out of 5.22 million school-aged children received treatment) [24]. As per the national population and housing census of 2012, the district had a population of about 203,597 people [25]. The dominant tribes in the district include Sukuma, Kurya and Jita. The main economic activities around the district include farming of both animal husbandry and crops, and fishing in the Lake Victoria.

School children aged 5–19 years from Nyamikoma village attending Fogofogo primary school participated in this study. The school/village is located approximately one kilometre from the Lake Victoria that serves as a main water source for all human needs in the village.

### Study design, sample size, and sampling procedures

This was a cross-sectional study nested within a longitudinal randomized clinical trial (registration number PACTR201612001914353) that was aimed at assessing the efficacy and safety of combination of praziquantel and artemisinin-based combination therapy on parasitological cure rate and egg reduction rate among *S*. *mansoni* infected school children.

Therefore, the sample size was calculated based on the WHO recommended formula [26] for calculating the number of children to be screened for evaluation of efficacy of antihelminthic drug based on the number of infected children required, compliance rate and prevalence of the disease. Six hundred schistosomiasis infected children were to be recruited in the longitudinal clinical trial. We used a prevalence of 85.6% reported by a recent study done among school children along the shores of Lake Victoria in a nearby region [6], and the compliance rate at 90%. The minimum sample size for the survey was calculated to be 830 school children.

One school (n = 1959) was purposively selected for the longitudinal randomized trial. School children from grades one to six were randomly selected for enrolment. In each grade, systematic random sampling was applied to enroll 130–140 children while keeping an equal number of the children in both sexes. After obtaining the sampling interval (K) and the starting point for each sex, every K^th^ child was sampled until the required number of children was reached. The sampling procedure was done carefully to ensure age and gender representation in the study were balanced.

### Inclusion and exclusion criteria

School children who reside/attend the selected village/school, whose parents/guardians gave informed consent for their participation, and who provided assent to participate were included in the study. Children who had a history of being treated with praziquantel within the past 6 months and/or antimalarial drug(s) e.g. Artemether-Lumefantrine (ALu) within the past 2 weeks before commencement of data collection were excluded.

### Data collection methods

A pre-tested structured questionnaire was used to collect socio-demographic information such as age (from school registry), gender, and grade, presenting symptoms, history of using praziquantel and/or antimalarial drugs and clinical data such as haemoglobin concentration and anthropometric measurements (body weight and height).

### Parasitological examination of intestinal schistosomiasis infection

Enrolled children were given information on the proper way to collect stool samples. Stool collection containers were given to children on the same day of sample collection. Two fresh stool samples were collected on two consecutive days from each enrolled participant. On each day, stool samples were collected at the temporary field site laboratory for processing. From each stool sample, two thick Kato Katz smears/slides were prepared using a 41.7 mg template [27]. The prepared Kato Katz smears/slides were transferred to NIMR Mwanza laboratory for examining the presence of schistosome’s eggs. The examination of the slides was done 24 hours after preparation to allow adequate coloration of the eggs for proper visibility under the microscope [27]. Microscopic examination of the slides was done by two trained and experienced technicians independently from NIMR Mwanza Research Centre laboratory. Other soil-transmitted helminths (STH) were not examined in this study.

A mean egg count from the four slides was calculated for each participant and recorded. The obtained mean egg counts were multiplied by 24 (constant factor) to obtain eggs count per gram of stool sample (epg) i.e. infection intensity for each participant. About 20% of the randomly selected Kato Katz slides were subject to quality assurance by an external technician who had been blinded of the results of the two study team technicians.

### Malaria diagnosis and haemoglobin concentration estimation

A finger prick blood was collected for malaria diagnosis and estimation of haemoglobin concentration. Malaria was diagnosed using SD BIOLINE Malaria Ag P.f/Pan MRDT (SD Standard Diagnostics, Inc, Korea), and results were recorded as either positive or negative. Haemoglobin concentration (in g/dL) was determined using HemoCue Hb 201+ machine (HemoCue AB Angelholm, Sweden) [28]. Presence or absence of anaemia was defined and categorized according to the WHO guideline; haemoglobin concentration < 11.5g/dL was considered as mild anaemia, haemoglobin concentration between 8.0 and 11.4g/dL was considered moderate anaemia, and haemoglobin concentration < 8.0g/dL was considered severe anaemia [29].

### Anthropometric measurements

Body weight was measured in kilogram (kg). The weighing scale was calibrated on a daily basis and body weight was measured to the nearest 0.1 kg. Height was measured in centimeter (cm). The anthropometric measurements were converted to height for age Z score (HAZ) and body mass index (BMI) for age Z score (BAZ) using WHO Anthro-Plus software version 1.0.4 for assessment of the nutritional status of the children [30]. Children whose HAZ and BAZ scores were less than 2 standard deviations were considered stunted and wasted/thin, respectively.

### Data management and statistical analysis

Data were double entered into the Census and Survey Processing System (CSPro) software (US Census Bureau, USA), cleaned and then analyzed using Statistical Package for Social Sciences (SPSS) for Windows version 20. Data analysis was done for 830 children who had complete data for intestinal schistosomiasis, stunting and thinness/wasting, and 824 children who had complete data for malaria and haemoglobin concentration. Infection intensity of each participant was calculated as an arithmetic mean of the four prepared thick Kato Katz smears. *Schistosoma mansoni* infection intensity (egg per gram of stool) was classified according to WHO classification [15] i.e. light infection (epg < 100), moderate infection (epg 100–399) and heavy infection (epg ≥ 400). Descriptive statistics was used to calculate the proportion of children infected with intestinal schistosomiasis, malaria, and undernutrition, the median infection intensity and haemoglobin concentrations of the studied population. Pearson’s chi-square test or Fishers exact test was used to compare proportions depending on the test appropriateness. Independent samples t-test was used to compare means between two groups. Wilcoxon-Mann-Whitney U-test was used to compare the median haemoglobin concentration between those with and without *Schistosoma mansoni* infection, age groups and sex. Negative binomial regression model was used to assess the predictors of eggs count/gram of stool among those infected. Univariate and multivariate logistic regression were used to identify predictors of intestinal schistosomiasis infection. Variables with p < 0.2 from the univariate analysis were included in the multivariate analysis model. Spearman correlation test was used to check the relationship between eggs intensity (epg) and haemoglobin concentration (g/dL). A *p-*value of < 0.05 was considered statistically significant.

### Ethical considerations

The study was approved by Institution Review Board of Muhimbili University of Health and Allied Sciences (MUHAS) (Ref:2016-5-25/AEC/Vol.X/03) and the Medical Research Coordination Committee (MRCC) of National Institute for Medical Research (NIMR), Tanzania (NIMR/HQ/R.8a/Vol.IX/2343). Before commencement of the study, permission to conduct the study was granted from all relevant Busega district authorities (District Executive Director (DED), District Medical Officer (DMO), District Education Officer (DEO), Village Leaders, School board and administration). Finally, written informed consent and assent from parents/guardians and children were obtained.

## Results

A total of 830 children were enrolled in the study. Nearly half of the participants 49.8% (413/830) were males. The mean age of the study participants was 11.7 years (±1.9 SD) and the median age was 12 years (7–19). At enrolment, 18.7% (155/830) of the enrolled children were complaining of pre-treatment abdominal pain. Table 1 summarizes the sociodemographic and baseline characteristics of the studied population.

**Table 1:** Sociodemographic and baseline characteristics of the studied population in Nyamikoma village.

Characteristic	Studied population N (%)	Schistosomiasis +ve N (%)	Schistosomiasis -ve N (%)
Age (years)			
Mean age ± SD	11.7 ± 1.9	11.6 ± 1.9	12.6 ± 2.0
≤ 12	575 (69.3)	536 (64.6)	39 (4.7)
>12	255 (30.7)	216 (26.0)	39 (4.7)
Sex			
Male	413 (49.8)	372 (44.8)	41 (4.9)
Female	417 (50.2)	380 (45.8)	37 (4.5)
** **Mean weight ± SD	30.9 ± 7.7	30.7 ± 7.4	33.2 ± 10.4
** **Mean height ± SD	137.1 ± 15.8	137.0 ± 14.4	138.1 ± 25.3
Abdominal pain			
Present	155 (18.7)	140 (16.9)	15 (1.8)
Absent	675 (81.3)	612 (73.7)	63 (7.6)
Stool consistency			
Formed	459 (55.3)	381 (45.9)	78 (9.4)
Soft	266 (32.0)	266 (32.0)	0 (0.0)
Loose	105 (12.7)	105 (12.7)	0 (0.0)

### Prevalence and intensity of intestinal schistosomiasis infection

Of the 830 screened children, 90.6% (752/830) were infected with intestinal schistosomiasis. Pre-adolescents (≤12 years) were more infected with intestinal schistosomiasis (93.2%) compared to adolescents (>12 years) (84.7%) (*χ*^*2*^ = 15.031, *p* < 0.001). There was no significant difference in the prevalence of infection between sex (*χ*^*2*^ = 0.271, *p* = 0.603). Children presenting with loose or soft stools at enrolment had a high prevalence of infection compared to those with formed stools (*χ*^*2*^ = 69.585, *p* < 0.0001). Having abdominal pain at enrollment was not associated with intestinal schistosomiasis infection (*χ*^*2*^ = 0.018 *p* = 0.89) **(**Table 2**)**. Though no significant association was found between abdominal pain and intensities of intestinal schistosomiasis infection (*χ*^*2*^ = 3.106, *p* = 0.37), abdominal pain was observed more in children with moderate (35.5%) and heavy infection (33.5%).

**Table 2:** Association of socio-demographic characteristics, malaria, anaemia and nutritional status with intestinal schistosomiasis infection among study participants.

Variable		Schistosomiasis +ve N (%)	Schistosomiasis–ve N (%)	*p*-value
Age (years)	≤12	536 (93.2)	39 (6.8)	< 0.001
	>12	216 (84.7)	39 (15.3)	
Sex	Male	372 (90.1)	41 (9.9)	0.60
	Female	380 (91.1)	37 (8.9)	
Malaria	Positive	13 (92.9)	1 (7.1)	0.13
	Negative	735 (90.7)	75 (9.3)	
Anaemia	Anaemic	181 (89.2)	22 (10.8)	0.36
	Not anaemic	567 (91.3)	54 (8.7)	
Abdominal pain	Present	140 (90.3)	15 (9.7)	0.89
	Absent	612 (90.7)	63 (9.3)	
Stool consistency	Loose	105 (100)	0 (0.0)	< 0.001
	Soft	266 (100)	0 (0.0)	
	Formed	381 (83.0)	78 (17.0)	
Stunting (HAZ)	Present	216 (89.6)	25 (10.4)	0.54
	Absent	536 (91.0)	53 (9.0)	
Wasting (BAZ)	Present	83 (88.3)	11 (11.7)	0.42
	Absent	669 (90.9)	67 (9.1)	

BAZ- body mass index (BMI) for age Z score, HAZ -height for age Z score

**Fig 1:**
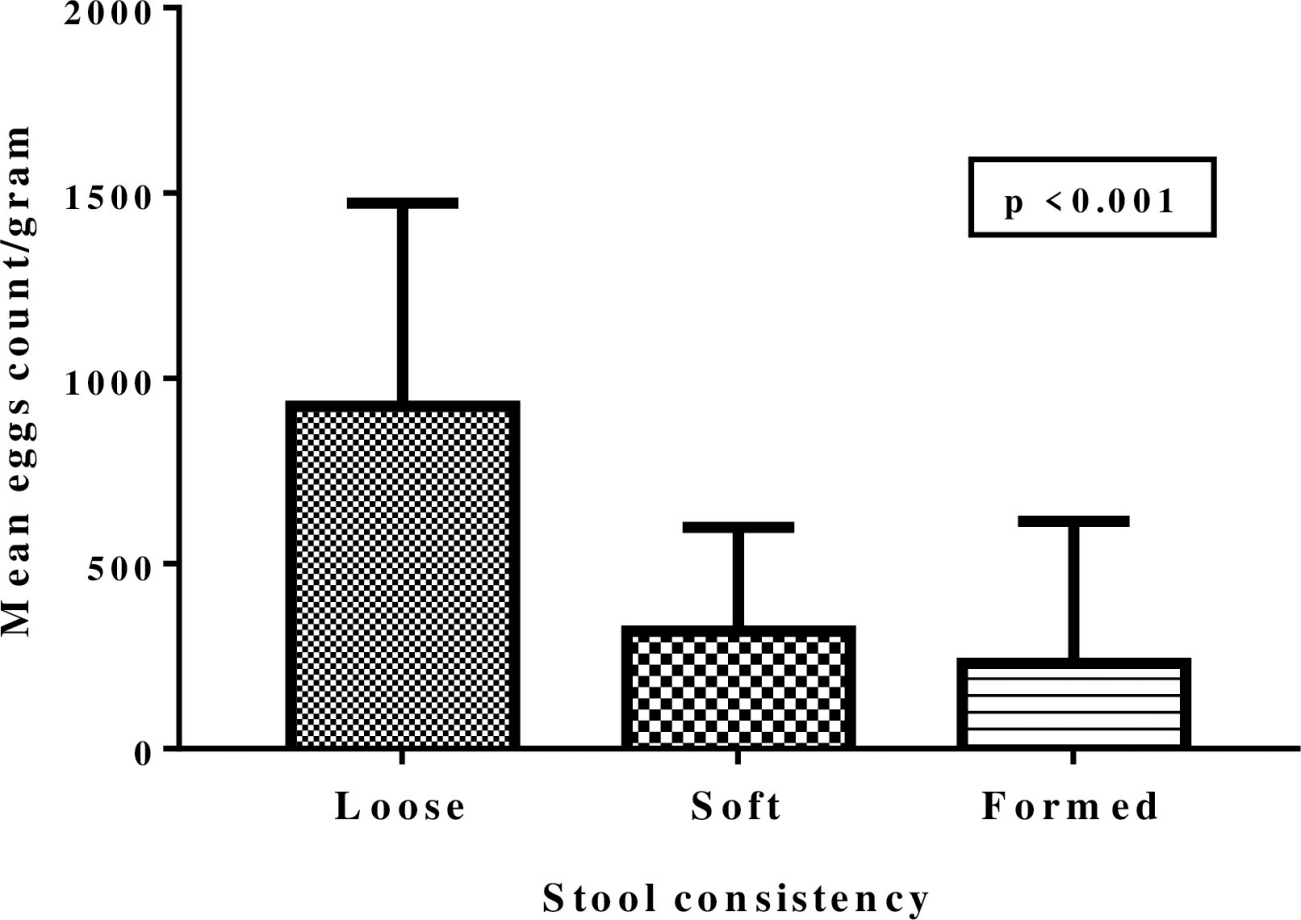
Infection intensity versus stool consistency. The Mean eggs count per gram of stool among children with loose stool was higher than in children with soft or formed stool.

The median infection intensity (epg) of the studied population was 204 (IQR 54–457). The distribution of infection intensities was as follows; 24.1% (200/830), 38.4% (319/830) and 28.1% (233/830) had a light, moderate and heavy infection, respectively. Children with loose stools were found to have higher mean eggs count per gram of stool compared to those with formed or soft stools (Fig 1). There was no statistically significant difference in the median infection intensity between age groups (*p* = 0.77). Though not significant (*χ*^*2*^ *=* 5.130, *p* = 0.16), boys had more heavy infections of intestinal schistosomiasis (54.9%) compared to girls (45.1%). However, on negative binomial regression model male sex, loose stool consistency, and stunting were significantly associated with high eggs count/gram of stool (*p* < 0.05). Deviance for goodness of model was 1.16 and Omnibus test *p* < 0.0001 (Table 3).

**Table 3:** Negative binomial regression model for predictors of eggs count/gram of stool among the study population.

	Parameter estimates				
Parameter	B	Std Error	95% (B)	Wald Chi-square	p-value
Constant	6.30	0.47	5.39–7.22	180.80	<0.0001
Age	-0.01	0.02	-0.03–0.05	0.33	0.56
Sex	-0.18	0.07	-0.32- -0.03	5.63	0.02
Anaemia	0.05	0.09	-0.12–0.21	0.28	0.59
Malaria	0.27	0.28	-0.28–0.82	0.95	0.33
Stool consistency	-0.50	0.05	-0.60- -0.41	111.83	<0.0001
Abdominal pain	-0.10	0.09	-0.28–0.08	1.18	0.28
Stunting (HAZ)	-0.31	0.08	-0.47- -0.14	13.29	<0.0001
Wasting (BAZ)	0.14	0.12	-0.09–0.37	1.51	0.22

### Prevalence of malaria, co-infection and association with intestinal schistosomiasis infection

Of the 824 participants tested for malaria, 1.7% (14/824) were infected with malaria. There was no significant difference in malaria prevalence between age groups (pre-adolescent and adolescent) (*χ*^*2*^ *=* 1.500, *p* = 0.48) and between sex (*χ*^*2*^ *=* 1.758, *p* = 0.46). Of the 14 children infected with malaria, 1.6% (13/824) were co-infected with intestinal schistosomiasis. The rate of co-infection among those with malaria was 92.8% (13/14). Of the 13 children with co-infection five had light, six had moderate, and two children had heavy intestinal schistosomiasis infection. There was no statistically significant association between intestinal schistosomiasis and malaria (*χ*^*2*^ = 3.675, *p* = 0.13).

### Prevalence of anaemia and association with intestinal schistosomiasis infection

**Fig 2:**
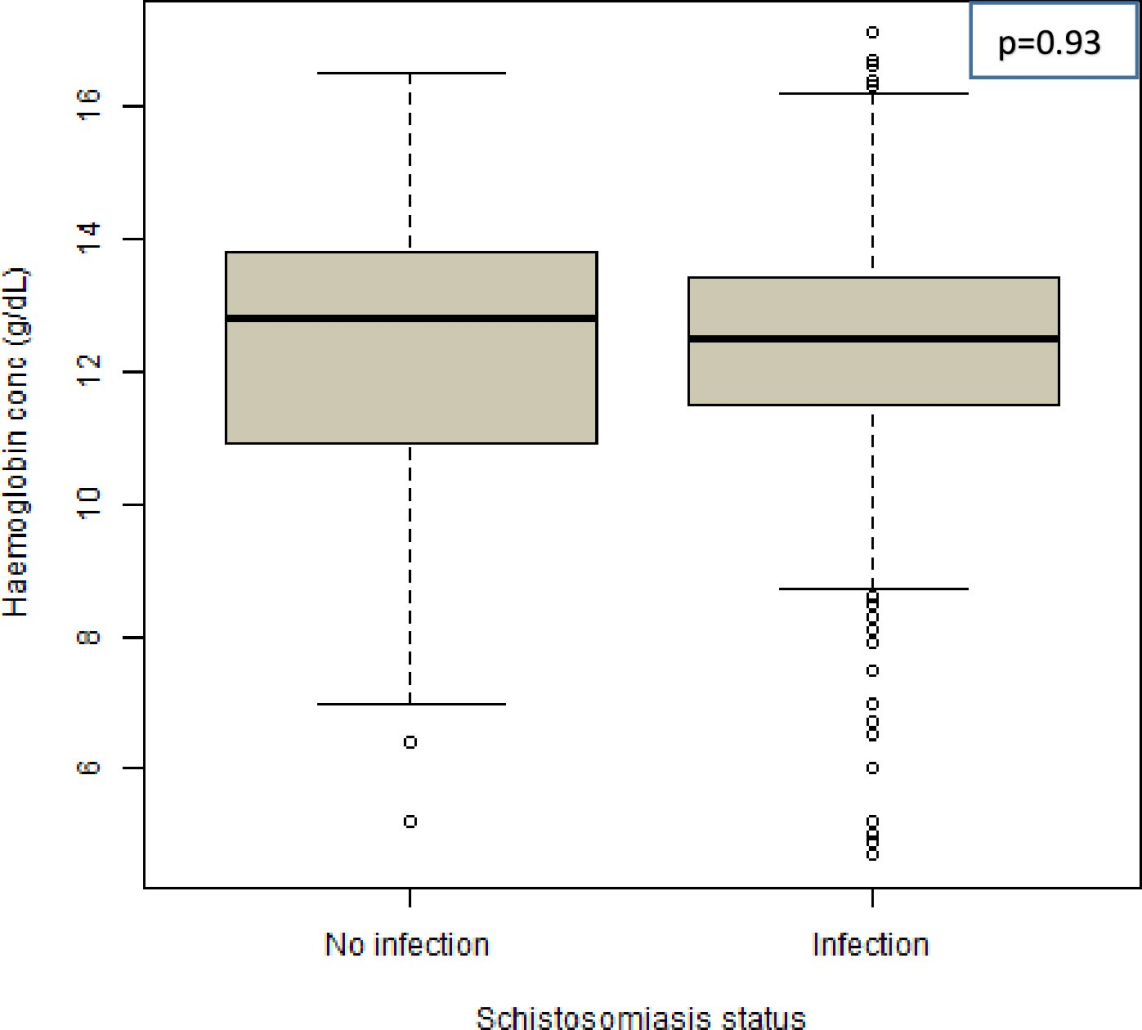
Haemoglobin concentration (g/dL) versus intestinal schistosomiasis infection. The median haemoglobin concentration (g/dL) of children with no intestinal schistosomiasis infection was slightly higher than children with intestinal schistosomiasis infection.

**Fig 3:**
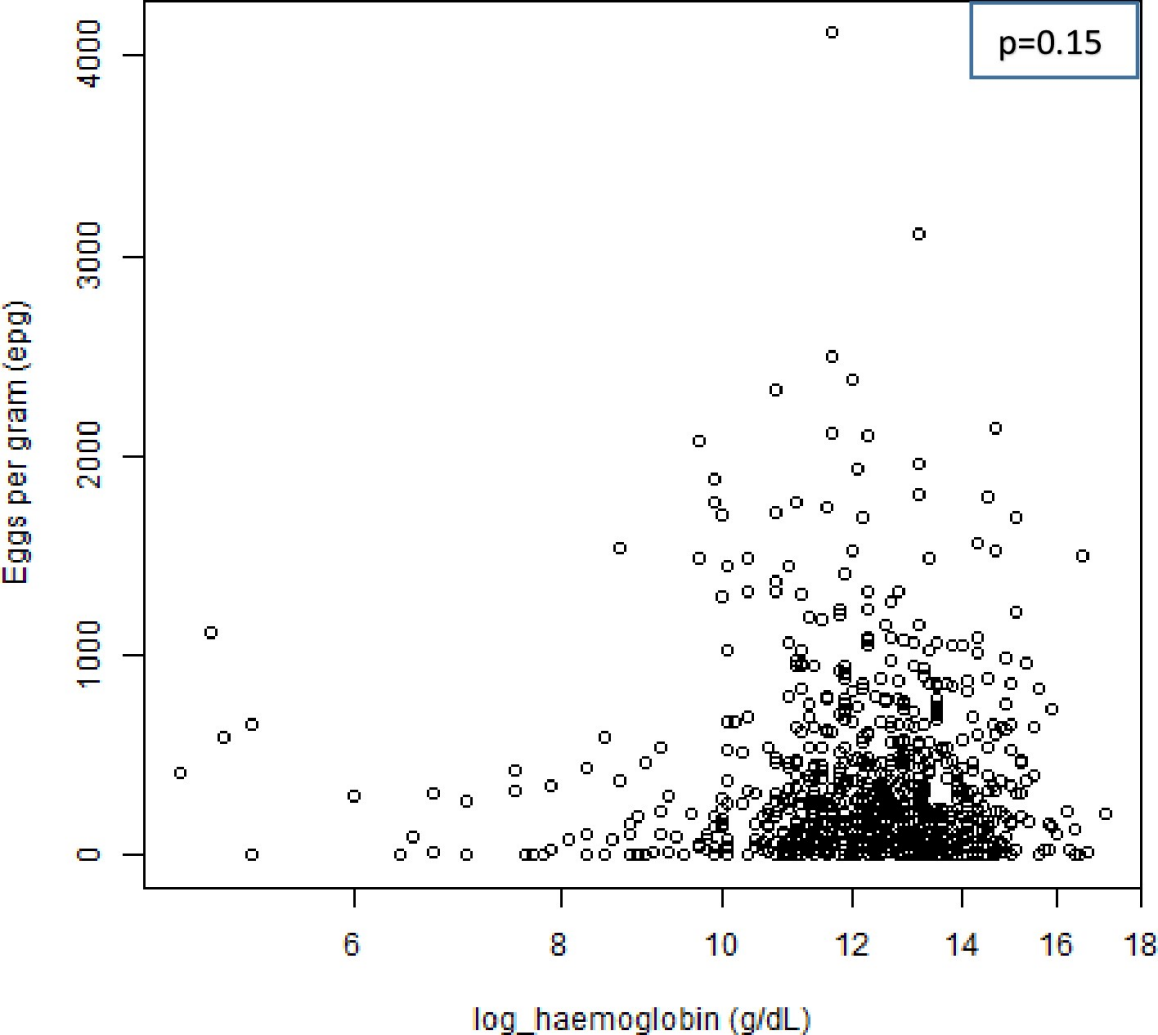
Scatter plot of eggs count per gram (epg) versus haemoglobin concentration (g/dL). A negative correlation between eggs count per gram of stool and haemoglobin concentration (Spearman correlation coefficient = -0.05).

Of the 824 screened children with complete data on haemoglobin concentration, 24.6% (203/824) were found to be anaemic. Mild, moderate and severe anaemia were 10.1% (83/824), 12.3% (101/824) and 2.3% (19/824), respectively. Out of the 19 children with severe anaemia, 10 children had moderate to heavy intestinal schistosomiasis infection. The median haemoglobin concentration of the studied population was 12.6 g/dL (IQR = 11.5–13.5g/dL). The median haemoglobin concentration was found to be higher in adolescent age group (13.0 g/dL) compared to pre-adolescent age group (12.4g/dL) (*p* < 0.001). There was no significant difference in the median haemoglobin concentration between sex (*p* = 0.38). Children who were not infected with intestinal schistosomiasis had slightly higher median haemoglobin concentration (12.8g/dL) compared with those who were infected (12.5g/dL) (Fig 2). There was no statistically significant association between intestinal schistosomiasis infection and anaemia (*χ*^*2*^ = 0.838, *p* = 0.36). There was a negative correlation between eggs count per gram of stool (epg) and haemoglobin concentration (Spearman correlation coefficient = - 0.05, p = 0.15 (Fig 3).

### Prevalence of undernutrition (stunting and wasting) and its association with intestinal schistosomiasis infection

Of the 830 screened children, 29.0% (241/830) were found to have stunting, and 11.3% (94/830) were found to be wasted. There was no statistically significant association between intestinal schistosomiasis infection and stunting (*χ*^*2*^ = 0.380, *p* = 0.53) or wasting (*χ*^*2*^ = 0.661, *p* = 0.41). However, on negative binomial regression model, stunting was found to be a significant predictor of high eggs count/gram of stool and not wasting (Table 3).

### Predictors of intestinal schistosomiasis infection

On univariate and multivariate logistic regression, lower age group was significantly associated with intestinal schistosomiasis infection. Age (years) and haemoglobin concentrations (g/dL) were included in the multivariate regression model (adjusted Odd Ratio model). The Hosmer and Lemeshow test for the goodness of fit for the multivariate analysis was a good fit (*χ*^*2*^ = 13.54, *p* = 0.09) (Table 4).

**Table 4:** Univariate and multivariate logistic regression analysis of the predictors of intestinal schistosomiasis in the studied population.

Variable		Schistosomiasis +ve N (%)	Univariate analysis			Multivariate analysis		
			cOR	95% CI	*p*-value	aOR	95% CI	*p*-value
Age (years)	≤ 12	536 (93.2)	2.48	1.54–3.97	< 0.001	2.43	1.51–3.92	<0.001
	>12	216 (84.7)	1^a^			1^a^		
Sex	Male	372 (90.1)	0.88	0.55–1.40	0.60	-	-	-
	Female	380 (91.1)	1^a^			-	-	-
Malaria	Positive	13 (92.9)	1.32	0.17–10.28	0.78	-	-	-
	Negative	735 (90.7)	1^a^			-	-	-
Anaemia status	Anaemic	181 (89.2)	0.78	0.46–1.32	0.36	-	-	-
	Not anaemic	567 (91.3)	1^a^			-	-	-
*Haemoglobin conc (g/dL)	-	-	1.09	0.96–1.25	0.16	1.11	0.98–1.26	0.10
Abdominal pain	Present	140 (90.3)	0.96	0.53–1.73	0.89	-	-	-
	Absent	612 (90.7)	1^a^			-	-	-
Stool consistency	Loose	105 (100)	3.3X10^8^			-	-	-
	Soft	266 (100)	3.3X10^8^			-	-	-
	Formed	381 (83.0)	1^a^			-	-	-
Stunting (HAZ)	Present	216 (89.6)	0.85	0.51–1.41	0.54	-	-	-
	Absent	536 (91.0)	1^a^			-	-	-
Wasting (BAZ)	Present	83 (88.3)	0.75	0.38–1.48	0.42	-	-	-
	Absent	669 (90.9)	1^a^			-	-	-

N-Total number of participants belong to the category

1^a:^ reference category, aOR: adjusted odds ratio, cOR: crude odds ratio

*—continuous variable

## Discussion

In the present study, we conducted a cross-sectional study to investigate the prevalence and intensity of intestinal schistosomiasis, malaria, anemia, malnutrition and combination thereof among school-aged children residing in Nyamikoma village in Busega district, Simiyu Region, north-western Tanzania. Our main findings indicate a high prevalence of intestinal schistosomiasis infection (90.6%) among school children in the study area despite five rounds of national mass praziquantel administration, anaemia (24.6%), stunting (29.0%) and wasting (11.3%), but a lower prevalence of malaria infection (1.7%) and malaria–intestinal schistosomiasis co-infection (1.6%) in the studied population.

The regular annual mass praziquantel treatment at the study area was interrupted in 2016 and this might have contributed to the observed high prevalence of the disease. Accordingly, skipping such important preventive chemotherapy in the area of high transmission for schistosomiasis may have a negative impact on the prevalence and intensity of the disease. Other factors that might have contributed to the observed high prevalence of intestinal schistosomiasis infection as reported by previous studies include close proximity of the school/village to the Lake [20, 31], lack of clean and safe water supply [32], poor sanitation and hygiene [33], and low knowledge about mode of transmission and prevention of the disease [34, 35]. Since the study area has the history of receiving repeated mass praziquantel treatment, poor efficacy of praziquantel to the immature (juvenile) stage of the parasite may be another reason to explain the continued and reported high prevalence of the disease around the study area [36]. After mass praziquantel treatment, the prevalence of schistosomiasis is expected to go down because the adults’ worms, which lay eggs, get killed by the drug and hence contamination of the environment decreases. Afterwards (mainly 4–6 weeks), juvenile worms grow and start laying eggs, contamination of the environment resume, and the prevalence goes up to the initial pre-treatment levels to continue transmission [1].

The observed prevalence of intestinal schistosomiasis in this study is higher than what was reported from previous studies conducted in other regions around the Lake in Northern Tanzania i.e. in Mara region (>80.0%) [6, 37] and Mwanza region (64.3%) [20]. The difference in prevalence between our study and other studies may be attributed to the focal nature or distribution of the disease. Another reason for the difference is that this study involved only one village, while the studies from other regions involved more than one village. The difference in the level of awareness, knowledge of disease transmission and prevention, and attitude towards the disease between the village residents may have contributed to the observed prevalence differences. On the other hand, our findings are similar to a recent study done in Madagascar where a higher prevalence of intestinal schistosomiasis infection (93.7%) was reported [38]. The slight difference observed in schistosomiasis prevalence between our study and a study done in Madagascar may be due to the different detection methods used i.e. Kato Katz technique versus Circulating Cathodic Antigen (CCA), respectively. A slightly lower prevalence (76.8%) was reported in Western Kenya using the same detection method i.e. Kato Katz technique [39].

The high prevalence of intestinal schistosomiasis infection found in the current study and in the previously reported studies indicate an urgent need for targeted interventions in addition to the current mass praziquantel treatment program for prevention and control of schistosomiasis around the Lake Zone and other endemic settings in the SSA region. Re-evaluation of the preventive chemotherapy being deployed by the National NTD Control Programs in SSA including the mode of delivery of annual mass praziquantel treatment such as giving repeated praziquantel doses may be considered [40] or research on alternative regimens such as combining praziquantel with artemisinin-based combination therapy targeting both the adult and juvenile parasites is recommended [41]. Apart from preventive chemotherapy, the provision of clean and safe water, improvement in sanitation and hygiene, and practical health education to school-aged children and the community is equally important in the study area.

The majority of the children had moderate (38.4%) to heavy (28.1%) schistosome infection as reflected by the observed high disease prevalence. This finding reflects a high exposure and transmission rate of the disease in the study area. The infection intensities observed may also reflect high level of contamination to the environment and the Lake that serves as the main source of water for all human needs in the study area. The observed infection intensity is contrary from that reported in Western Kenya where the majority of their participants had light (35.5%) to moderate infection (25.2%) [31, 39].

In this study, pre-adolescents were found to be more infected than adolescents. The pre-adolescents may have low immunity compared with the adolescents thus contributing to more susceptibility to infection. We also speculate that the observed high prevalence of the disease in the pre-adolescents may be a carry-over since the pre-school children are not part of the mass praziquantel treatment campaigns in Tanzania. The findings are similar to studies conducted in other localities around the Lake Victoria basin [32]. However, our results were different from reports from other studies where age was not significantly associated with intestinal schistosomiasis infection [39].

The present study has found no sex difference in the prevalence of intestinal schistosomiasis infection, although males (54.9%) were found to be more heavily infected than females (45.1%). Both boys and girls may be exposed to the same contaminated environment in the village/school and the Lake thus contributing to the same level of infection. A similar finding has been reported in Western Kenya [39]. Stool consistency was found to be associated with intestinal schistosomiasis infection and a significant predictor of eggs count/gram of stool among those infected. Children with loose stool were found to be more and heavily infected than those with soft or formed stool.

Our study has found a very low prevalence of malaria (< 2%) among the screened children in the study area. The observed low prevalence of malaria was probably due to the ongoing research on the effect of indoor residual spraying (IRS) around the study area, which started before data collection for this study has commenced. The finding indicates the importance of effective IRS in preventing malaria transmission together with other measures such as use of long-lasting insecticide-treated nets and effective case management.

Since the prevalence of malaria was found to be very low, and the association between intestinal schistosomiasis and malaria infection did not reach significance. The co-infection rate of malaria and intestinal schistosomiasis in the studied population was very low (1.6% of the screened children had malaria-*S*. *mansoni* co-infection). However, the rate of co-infection for those who had malaria was very high 92.8% (13 out of 14 malaria cases). This might be due to a high burden of intestinal schistosomiasis observed in the study area. Previous studies done in Tanzania [6, 20] and in Uganda [42] reported an average of 20–25%, and 13.1% in Kenya [39] of co-infection in the studied populations but a similarly high rate of co-infection among those with malaria [6]. On the other hand, the rate of co-infection observed in this study was similar to a study done in the Democratic Republic of Congo where a co-infection prevalence of 1.5% was reported [43]. Our study did not found a significant association between intestinal schistosomiasis and malaria contrary to report from other studies [44]. It has been reported that *Schistosoma mansoni* infection affects the immunological protection of malaria by changing Th1 and Th2 immune responses thus increase susceptibility to malaria [45–47]. The insignificant association found in this study may be contributed by the small number of children who were co-infected with malaria and intestinal schistosomiasis infection.

In this study, about one-quarter of the screened children (24.6%) were found to have anaemia, and the majority of them had mild to moderate anaemia. The observed burden of anaemia among school children may be greatly attributed to poor nutrition among the study participants in the study area. The findings are similar to the ones reported within the country by Munisi *et al* [48] where a prevalence of anaemia of 29.4% was reported. Other studies; Tanzania (34.4%) and Angola (> 40%) reported a higher prevalence of anaemia compared to our study [49, 50]. There was no significant association found between intestinal schistosomiasis infection and anaemia, similar to the findings reported by other studies done in African children [48, 51]. The prevalence of anaemia may also depend on food availability, endemicity of other parasitic infections such as malaria and hookworms in the regions.

In the present study, more than a quarter of the screened children (29.0%) were found to have stunted growth. Since there was no significant association found between intestinal schistosomiasis infection and stunting in this study, stunting may be a consequence of poor nutrition to the children. However, it was found that stunting significantly predicts infection intensity among those infected. Previous study done in North-Western of Tanzania [48] has reported a much higher prevalence of stunting of 38.2% among school children. A similar finding has been also reported in Northern Angola (32.2%), despite the differences in diet, culture and environment between populations studied [49]. A study done in the Mara region of Tanzania reported a slightly lower prevalence of stunting (21.6%) [6]. On the other hand, 11.3% of the children were found to have wasting. Wasting was not significantly associated with intestinal schistosomiasis infection or intensity. A study done in the North–Western zone of Tanzania reported a slightly higher prevalence of wasting of 14.4% compared to our study [48]. As explained above, these forms of undernutrition may be greatly attributed to poor nutrition to the children as elucidated by others [52].

## Conclusions

We conclude that intestinal schistosomiasis is still highly prevalent among school children in the study area despite the ongoing national mass praziquantel treatments. Prevalence of malaria was found to be very low, but undernutrition and anaemia continues to be a burden among school children. Although there is a need for further research (i.e. randomized clinical trials) on preventive and control measures to address schistosomiasis burden in Tanzania, provision of clean and safe water, improvement in sanitation and hygiene, practical health education to school children is equally important. The mode of delivery of mass praziquantel treatments needs re-evaluation to look for the most effective way or alternative regimens to achieve disease control.

## Supporting information

S1 Checklist (PDF)

## References

[1] Adenowo AF, Oyinloye BE, Ogunyinka BI, Kappo AP. Impact of human schistosomiasis in sub-Saharan Africa. Braz J Infect Dis. 2015;19(2):196–205. Epub 2015/02/01. 10.1016/j.bjid.2014.11.004 . 25636189 PMC9425372

[2] Utzinger J, Raso G, Brooker S, De Savigny D, Tanner M, Ornbjerg N, et al Schistosomiasis and neglected tropical diseases: towards integrated and sustainable control and a word of caution. Parasitology. 2009;136(13):1859–74. Epub 2009/11/13. 10.1017/S0031182009991600 19906318 PMC2791839

[3] Steinmann P, Keiser J, Bos R, Tanner M, Utzinger J. Schistosomiasis and water resources development: systematic review, meta-analysis, and estimates of people at risk. Lancet Infect Dis. 2006;6(7):411–25. Epub 2006/06/23. 10.1016/S1473-3099(06)70521-7 . 16790382

[4] Mazigo HD, Nuwaha F, Kinung'hi SM, Morona D, Pinot de Moira A, Wilson S, et al Epidemiology and control of human schistosomiasis in Tanzania. Parasites & vectors. 2012;5:274 Epub 2012/11/30. 10.1186/1756-3305-5-274 23192005 PMC3549774

[5] Hotez PJ, Fenwick A, Savioli L, Molyneux DH. Rescuing the bottom billion through control of neglected tropical diseases. Lancet (London, England). 2009;373(9674):1570–5. Epub 2009/05/05. 10.1016/s0140-6736(09)60233-6 . 19410718

[6] Kinung'hi SM, Mazigo HD, Dunne DW, Kepha S, Kaatano G, Kishamawe C, et al Coinfection of intestinal schistosomiasis and malaria and association with haemoglobin levels and nutritional status in school children in Mara region, Northwestern Tanzania: a cross-sectional exploratory study. BMC research notes. 2017;10(1):583 Epub 2017/11/11. 10.1186/s13104-017-2904-2 29121978 PMC5679344

[7] Hotez PJ, Kamath A. Neglected tropical diseases in sub-saharan Africa: review of their prevalence, distribution, and disease burden. PLoS neglected tropical diseases. 2009;3(8):e412 Epub 2009/08/27. 10.1371/journal.pntd.0000412 19707588 PMC2727001

[8] Bangert M, Molyneux DH, Lindsay SW, Fitzpatrick C, Engels D. The cross-cutting contribution of the end of neglected tropical diseases to the sustainable development goals. Infectious diseases of poverty. 2017;6(1):73 Epub 2017/04/05. 10.1186/s40249-017-0288-0 28372566 PMC5379574

[9] Molyneux DH, Hotez PJ, Fenwick A. "Rapid-impact interventions": how a policy of integrated control for Africa's neglected tropical diseases could benefit the poor. PLoS medicine. 2005;2(11):e336 Epub 2005/10/11. 10.1371/journal.pmed.0020336 16212468 PMC1253619

[10] Fiona Samuels and Romina Rodríguez Pose. Why neglected tropical diseases matter in reducing poverty, developmentprogress.org, July 2013.

[11] Baird S, Hicks JH, Kremer M, Miguel E. Worms at Work: Long-run Impacts of a Child Health Investment. The quarterly journal of economics. 2016;131(4):1637–80. Epub 2016/11/08. 10.1093/qje/qjw022 27818531 PMC5094294

[12] Brolan CE, Te V, Floden N, Hill PS, Forman L. Did the right to health get across the line? Examining the United Nations resolution on the Sustainable Development Goals. BMJ global health. 2017;2(3):e000353 Epub 2017/12/12. 10.1136/bmjgh-2017-000353 29225946 PMC5717936

[13] Tchuem Tchuente LA, Rollinson D, Stothard JR, Molyneux D. Moving from control to elimination of schistosomiasis in sub-Saharan Africa: time to change and adapt strategies. Infectious diseases of poverty. 2017;6(1):42 Epub 2017/02/22. 10.1186/s40249-017-0256-8 28219412 PMC5319063

[14] Hotez PJ, Herricks JR. Helminth elimination in the pursuit of sustainable development goals: a "worm index" for human development. PLoS neglected tropical diseases. 2015;9(4):e0003618 Epub 2015/05/01. 10.1371/journal.pntd.0003618 25928617 PMC4415765

[15] WHO. Schistosomiasis: progress report 2001–2011, strategic plan 2012–2020. World Health Organization: Geneva, Switzerland, 2013.

[16] WHO. World malaria report. World Health Organization: Geneva, Switzerland, 2017.

[17] Nankabirwa J, Brooker SJ, Clarke SE, Fernando D, Gitonga CW, Schellenberg D, et al Malaria in school-age children in Africa: an increasingly important challenge. Tropical medicine & international health: TM & IH. 2014;19(11):1294–309. Epub 2014/08/26. 10.1111/tmi.12374 25145389 PMC4285305

[18] Ministry of Health CD, Gender, Elderly and Children (MoHCDGEC) [Tanzania Mainland], Ministry of Health (MoH) [Zanzibar], National Bureau of Statistics (NBS), Office of the Chief Government Statistician (OCGS), and ICF, 2016 Tanzania Demographic and Health Survey and Malaria Indicator Survey (TDHS-MIS). Dar es Salaam, Tanzania, and Rockville, Maryland, USA: MoHCDGEC, MoH, NBS, OCGS, and ICF: 2015–2016.

[19] Booth M. The role of residential location in apparent helminth and malaria associations. Trends in parasitology. 2006;22(8):359–62. Epub 2006/06/27. 10.1016/j.pt.2006.06.007 . 16797235

[20] Mazigo HD, Waihenya R, Lwambo NJ, Mnyone LL, Mahande AM, Seni J, et al Co-infections with Plasmodium falciparum, Schistosoma mansoni and intestinal helminths among schoolchildren in endemic areas of northwestern Tanzania. Parasites & vectors. 2010;3:44 Epub 2010/05/21. 10.1186/1756-3305-3-44 20482866 PMC2881914

[21] Simon GG. Impacts of neglected tropical disease on incidence and progression of HIV/AIDS, tuberculosis, and malaria: scientific links. International journal of infectious diseases: IJID: official publication of the International Society for Infectious Diseases. 2016;42:54–7. Epub 2015/11/26. 10.1016/j.ijid.2015.11.006 . 26594012

[22] Ahmed AM, Abbas H, Mansour FA, Gasim GI, Adam I. Schistosoma haematobium infections among schoolchildren in central Sudan one year after treatment with praziquantel. Parasites & vectors. 2012;5:108 Epub 2012/06/09. 10.1186/1756-3305-5-108 22676052 PMC3434009

[23] Garba A, Lamine MS, Barkire N, Djibo A, Sofo B, Gouvras AN, et al Efficacy and safety of two closely spaced doses of praziquantel against Schistosoma haematobium and S. mansoni and re-infection patterns in school-aged children in Niger. Acta tropica. 2013;128(2):334–44. Epub 2012/09/04. 10.1016/j.actatropica.2012.08.008 . 22940014

[24] The Ministry of Health Community development, Gender, Elderly and Children (MoHCDEC). The United Republic of Tanzania. Uniting to combat Neglected Tropical Diseases; Mass treatment coverage for NTDs. United Republic of Tanzania and neglected tropical diseases. https://unitingtocombatntds.org/wp-content/uploads/2018/01/Tanzania_eng.pdf: 2016.

[25] National Bureau of Statistics (NBS). Population and housing census. Ministry of Finance, Dar es Salaam, The United Republic of Tanzania: 2012.

[26] World Health Organization. Assessing the efficacy of anthelminthic drugs against schistosomiasis and soil-transmitted helminthiases. World Health Organization, 2013.

[27] WHO. Basic Laboratory Methods in Medical Parasitology. World Health Organization, Geneva, Switzerland: 1991.

[28] Nkrumah B, Nguah SB, Sarpong N, Dekker D, Idriss A, May J, et al Hemoglobin estimation by the HemoCue(R) portable hemoglobin photometer in a resource poor setting. BMC clinical pathology. 2011;11:5 Epub 2011/04/23. 10.1186/1472-6890-11-5 21510885 PMC3095531

[29] World Health Organization. Haemoglobin concentrations for the diagnosis of anaemia and assessment of severity. 2011.

[30] WHO. WHO Anthroplus Software; Software for assessing Growth and Development of the World's Children and Adolescents. Department of Nutrition for Health and Development, World Health Organization, Geneva, Switzerland, 2009.

[31] Odiere MR, Rawago FO, Ombok M, Secor WE, Karanja DM, Mwinzi PN, et al High prevalence of schistosomiasis in Mbita and its adjacent islands of Lake Victoria, western Kenya. Parasites & vectors. 2012;5:278 Epub 2012/12/05. 10.1186/1756-3305-5-278 23206383 PMC3523971

[32] Mugono M, Konje E, Kuhn S, Mpogoro FJ, Morona D, Mazigo HD. Intestinal schistosomiasis and geohelminths of Ukara Island, North-Western Tanzania: prevalence, intensity of infection and associated risk factors among school children. Parasites & vectors. 2014;7:612 Epub 2014/12/24. 10.1186/s13071-014-0612-5 25533267 PMC4297386

[33] Kabatereine N, Fleming F, Thuo W, Tinkitina B, Tukahebwa EM, Fenwick A. Community perceptions, attitude, practices and treatment seeking behaviour for schistosomiasis in L. Victoria islands in Uganda. BMC research notes. 2014;7:900 Epub 2014/12/17. 10.1186/1756-0500-7-900 25495121 PMC4307169

[34] Odhiambo GO, Musuva RM, Atuncha VO, Mutete ET, Odiere MR, Onyango RO, et al Low levels of awareness despite high prevalence of schistosomiasis among communities in Nyalenda informal settlement, Kisumu city, western Kenya. PLoS neglected tropical diseases. 2014;8(4):e2784 Epub 2014/04/05. 10.1371/journal.pntd.0002784 24699502 PMC3974654

[35] Munisi DZ, Buza J, Mpolya EA, Angelo T, Kinung'hi SM. Knowledge, attitude, and practices on intestinal schistosomiasis among primary schoolchildren in the Lake Victoria basin, Rorya District, north-western Tanzania. BMC public health. 2017;17(1):731 Epub 2017/09/25. 10.1186/s12889-017-4767-9 28934944 PMC5609045

[36] Inobaya MT, Olveda RM, Chau TN, Olveda DU, Ross AG. Prevention and control of schistosomiasis: a current perspective. Research and reports in tropical medicine. 2014;2014(5):65–75. Epub 2014/11/18. 10.2147/RRTM.S44274 25400499 PMC4231879

[37] Munisi DZ, Buza J, Mpolya EA, Kinung'hi SM. Intestinal Schistosomiasis among Primary Schoolchildren in Two On-Shore Communities in Rorya District, Northwestern Tanzania: Prevalence, Intensity of Infection and Associated Risk Factors. Journal of parasitology research. 2016;2016:1859737 Epub 2016/11/09. 10.1155/2016/1859737 27822385 PMC5086394

[38] Spencer SA, Penney J, Russell HJ, Howe AP, Linder C, Rakotomampianina ALD, et al High burden of Schistosoma mansoni infection in school-aged children in Marolambo District, Madagascar. Parasites & vectors. 2017;10(1):307 Epub 2017/06/26. 10.1186/s13071-017-2249-7 28646926 PMC5483300

[39] Nagi S, Chadeka EA, Sunahara T, Mutungi F, Justin YK, Kaneko S, et al Risk factors and spatial distribution of Schistosoma mansoni infection among primary school children in Mbita District, Western Kenya. PLoS neglected tropical diseases. 2014;8(7):e2991 Epub 2014/07/25. 10.1371/journal.pntd.0002991 25058653 PMC4109881

[40] Kabuyaya M, Chimbari MJ, Mukaratirwa S. Efficacy of praziquantel treatment regimens in pre-school and school aged children infected with schistosomiasis in sub-Saharan Africa: a systematic review. Infectious diseases of poverty. 2018;7(1):73 Epub 2018/07/11. 10.1186/s40249-018-0448-x 29986763 PMC6036702

[41] Bergquist R, Elmorshedy H. Artemether and Praziquantel: Origin, Mode of Action, Impact, and Suggested Application for Effective Control of Human Schistosomiasis. Tropical medicine and infectious disease. 2018;3(4). Epub 2018/12/24. 10.3390/tropicalmed3040125 30572592 PMC6306701

[42] Kabatereine NB, Standley CJ, Sousa-Figueiredo JC, Fleming FM, Stothard JR, Talisuna A, et al Integrated prevalence mapping of schistosomiasis, soil-transmitted helminthiasis and malaria in lakeside and island communities in Lake Victoria, Uganda. Parasites & vectors. 2011;4:232 Epub 2011/12/15. 10.1186/1756-3305-4-232 22166365 PMC3270004

[43] Matangila JR, Doua JY, Linsuke S, Madinga J, Inocencio da Luz R, Van Geertruyden JP, et al Malaria, schistosomiasis and soil transmitted helminth burden and their correlation with anemia in children attending primary schools in Kinshasa, Democratic Republic of Congo. PloS one. 2014;9(11):e110789 Epub 2014/11/06. 10.1371/journal.pone.0110789 25372029 PMC4220949

[44] Ndeffo Mbah ML, Skrip L, Greenhalgh S, Hotez P, Galvani AP. Impact of Schistosoma mansoni on malaria transmission in Sub-Saharan Africa. PLoS neglected tropical diseases. 2014;8(10):e3234 Epub 2014/10/21. 10.1371/journal.pntd.0003234 25329403 PMC4199517

[45] Maizels RM, Pearce EJ, Artis D, Yazdanbakhsh M, Wynn TA. Regulation of pathogenesis and immunity in helminth infections. The Journal of experimental medicine. 2009;206(10):2059–66. Epub 2009/09/23. 10.1084/jem.20091903 19770272 PMC2757871

[46] Su Z, Segura M, Morgan K, Loredo-Osti JC, Stevenson MM. Impairment of protective immunity to blood-stage malaria by concurrent nematode infection. Infection and immunity. 2005;73(6):3531–9. Epub 2005/05/24. 10.1128/IAI.73.6.3531-3539.2005 15908382 PMC1111846

[47] Osada Y, Kanazawa T. Schistosome: its benefit and harm in patients suffering from concomitant diseases. Journal of biomedicine & biotechnology. 2011;2011:264173 Epub 2011/12/02. 10.1155/2011/264173 22131800 PMC3216407

[48] Munisi DZ, Buza J, Mpolya EA, Kinung'hi SM. Schistosoma mansoni Infections, Undernutrition and Anaemia among Primary Schoolchildren in Two Onshore Villages in Rorya District, North-Western Tanzania. PloS one. 2016;11(12):e0167122 Epub 2016/12/10. 10.1371/journal.pone.0167122 27936031 PMC5147845

[49] Sousa-Figueiredo JC, Gamboa D, Pedro JM, Fancony C, Langa AJ, Magalhaes RJ, et al Epidemiology of malaria, schistosomiasis, geohelminths, anemia and malnutrition in the context of a demographic surveillance system in northern Angola. PloS one. 2012;7(4):e33189 Epub 2012/04/12. 10.1371/journal.pone.0033189 22493664 PMC3320883

[50] Kinung'hi SM, Magnussen P, Kaatano GM, Kishamawe C, Vennervald BJ. Malaria and helminth co-infections in school and preschool children: a cross-sectional study in Magu district, north-western Tanzania. PloS one. 2014;9(1):e86510 Epub 2014/02/04. 10.1371/journal.pone.0086510 24489732 PMC3906044

[51] Mboera LE, Senkoro KP, Rumisha SF, Mayala BK, Shayo EH, Mlozi MR. Plasmodium falciparum and helminth coinfections among schoolchildren in relation to agro-ecosystems in Mvomero District, Tanzania. Acta tropica. 2011;120(1–2):95–102. Epub 2011/07/12. 10.1016/j.actatropica.2011.06.007 . 21741929

[52] Mohamed I, Kinung'hi S, Mwinzi PNM, Onkanga IO, Andiego K, Muchiri G, et al Diet and hygiene practices influence morbidity in schoolchildren living in Schistosomiasis endemic areas along Lake Victoria in Kenya and Tanzania-A cross-sectional study. PLoS neglected tropical diseases. 2018;12(3):e0006373 Epub 2018/03/29. 10.1371/journal.pntd.0006373 29590175 PMC5891076

