# Supplementary material for: Prevalence and correlates of intestinal schistosomiasis infection among school-aged children in North-Western Tanzania
Source: PLoS One. 2020 Feb 5;15(2):e0228770. doi: 10.1371/journal.pone.0228770 (PMC7001966; doi:10.1371/journal.pone.0228770)
Supplement: S1 Checklist — (PDF) [file pone.0228770.s001.pdf]

# OPTIMIZATION OF SCHISTOSOMIASIS TREATMENT IN TANZANIA

**CONFIDENTIAL CASE RECORD FORM**
**STUDY SITE: PRIMARY SCHOOL .....................DISTRICT: .............................., TANZANIA**

**SUBJECT NAME: ..................**
**SUBJECT SCREENING ID: .......................**
**SUBJECT STUDY ID: ..........................**

**SCREENING VISIT:**

Date interviewed for screening (dd/mm/yy): ........./........../............

| **Criteria for screening** – please tick the appropriate boxes**:** | | |
|---|---|---|
| 1) Signed Informed Consent form (Parent, Guardian, or child)<br>Consent date (dd/mm/yy): ........../.........../............ | ☐ Y | ☐ N |
| 2) Able and willing to be followed-up for 2 months | ☐ Y | ☐ N |
| 3) Age: 6 -18 years | ☐ Y | ☐ N |

***If both answers are YES, proceed:***

**DEMOGRAPHICS**

| | |
|---|---|
| Age: ______ years | Sex: ☐M ☐F |
| School: ______ | Class/Grade: ______ |
| | |
| **Inclusion criteria for screening – please tick the appropriate boxes:** | |

***If all answers are YES, proceed:***

| | | |
|---|---|---|
| 5) Experiencing a severe concurrent medical condition e.g. SCD | ☐ Y | ☐ N |
| 6) History of praziquantel use within previous 6 months | ☐ Y | ☐ N |
| 7)Have diarrhea at time of the first sampling | ☐ Y | ☐ N |

***If all answers are NO, proceed:***

Karolinska Institutet

MUHAS

# OPTIMIZATION OF SCHISTOSOMIASIS TREATMENT IN TANZANIA

***SAMPLE COLLECTION AND RESULTS AT SCREENING.***

**Stools exams** ☐**Yes** ☐**No** **NA** ☐

Date of stool sample collection (dd/mm/yy) : ___/___/___

Consistency of stools: ☐ formed ☐ soft ☐ loose ☐ watery
☐ blood ☐ mucus

Result of parasitological exam for *S. Mansoni* (Kato Katz):
Slide 1: _______ eggs; Slide 2: _______ eggs; Slide 3: _______ eggs; Slide 4: _______ eggs

Result of parasitological exam for *Hookworm*(Kato Katz):
Slide 1: _______ eggs; Slide 2: _______ eggs; Slide 3: _______ eggs; Slide 4: _______ eggs

Result of parasitological exam for *A.lumbricoides* (Kato Katz):
Slide 1: _______ eggs; Slide 2: _______ eggs; Slide 3: _______ eggs; Slide 4: _______ eggs

Result of parasitological exam for *T.trichiura* (Kato Katz):
Slide 1: _______ eggs; Slide 2: _______ eggs; Slide 3: _______ eggs; Slide 4: _______ eggs

Egg count (*S.mansoni*) at screening: _______ eggs= _____eggs/gram of stool

**Infection intensity: Light ☐ moderate ☐ Heavy ☐**

***Quality control (on 10% of slides)***
***To be completed by the senior lab technician***

***Egg count at screening: _______ eggs= _____eggs/gram of stool***

**Urine exams** ☐**Yes** ☐**No** **NA** ☐

Date of urine sample collection (dd/mm/yy) : ___/___/___

Consistency/colouration of urine: ☐ turbidity ☐ Reddish brown ☐ bloody
☐ yellowish ☐clear/colourless

Result of parasitological exam (urine filtration):
Slide 1: _______ eggs; Slide 2: _______ eggs; Slide 3: _______ eggs;
Slide 4: _______ eggs

**Egg count (*S.haematobium*) at screening: _______ eggs= _____eggs/per 10mls of urine**

**Infection intensity: Light ☐ moderate ☐ Heavy ☐**

Karolinska Institutet

MUHAS

# OPTIMIZATION OF SCHISTOSOMIASIS TREATMENT IN TANZANIA

***Quality control (on 10% of slides)***
***To be completed by the senior lab technician***

***Egg count at screening: ________ eggs= ______eggs/per 10mls of urine***

**Is urine been kept for miracidia collection? ☐Yes ☐No**

Is the patient eligible for enrolment? ☐Yes ☐No

______________________________
Study team person signature

Date (dd/mm/yy): ................................

Karolinska Institutet

MUHAS

# OPTIMIZATION OF SCHISTOSOMIASIS TREATMENT IN TANZANIA

**CONFIDENTIAL CASE RECORD FORM**
**STUDY SITE: PRIMARY SCHOOL ......................DISTRICT: .............................., TANZANIA**

**SUBJECT NAME: .............................................**
**SUBJECT SCREENING ID: ...............................................**
**SUBJECT STUDY ID: ....................................................**

**SCREENING VISIT: CLINICAL SYMPTOMS, MALARIA, HAEMOGLOBIN CONCENTRATION ANTHROPOMETRIC MEASUREMENTS**

Date interviewed for screening (dd/mm/yy): ........./........../............

**PATIENT INFORMATION:**

WEIGHT [ Kgs ]

HEIGHT [ cm ]

Any history of using antimalarial in the past 2 weeks
YES [ ] NO [ ]

Any history of one of the following symptoms in the past 3 days

FEVER: YES [ ] NO [ ]

HEADACHE: YES [ ] NO [ ]

VOMITING: YES [ ] NO [ ]

FATIGUE: YES [ ] NO [ ]

Haemoglobin level [ g/dl ]

Blood slide for malaria parasite taken YES [ ] NO [ ]

RESULTS: .........................................................................

Karolinska Institutet

MUHAS
